# Collagen Type IV Alpha 5 Chain in Bronchiolitis Obliterans Syndrome After Lung Transplant: The First Evidence

**DOI:** 10.1007/s00408-023-00632-8

**Published:** 2023-07-04

**Authors:** M. Armati, S. Cattelan, M. Guerrieri, M. Messina, B. Perea, M. Genovese, M. d’Alessandro, S. Gangi, P. Cameli, F. Perillo, D. Bennett, A. Fossi, Tuscany Transplant Group, E. Bargagli, L. Bergantini

**Affiliations:** 1 Department of Medical Sciences, Surgery and Neurosciences, Respiratory Disease and Lung Transplant Unit, Siena University, 53100 Siena, Italy; 2 Unit of Respiratory Diseases, Department of Medicine, Surgery and Pharmacy, University of Sassari, 07100 Sassari, Italy; 3 Thoracic Surgery Unit, Department of Experimental and Clinical Medicine, Azienda Ospedaliero-Universitaria Careggi, 50134 Florence, Italy; 4 Anesthesia and Intensive Care Unit, University Hospital of Siena, Siena, Italy; 5 Lung Transplant Unit, Department of Medical, Surgical and Neuro Sciences, Azienda Ospedaliero-Universitaria Senese, University of Siena, 53100 Siena, Italy; 6 Organizzazione Toscana Trapianti, Regione Toscana, 50134 Florence, Italy

**Keywords:** COL4A5, Biomarker, BOS, LTX

## Abstract

**Introduction:**

Bronchiolitis obliterans syndrome (BOS) is the most common form of CLAD and is characterized by airflow limitation and an obstructive spirometry pattern without parenchymal opacities. The protein signature of BOS lesions concerns extracellular matrix organization and aberrant basement membrane composition. In this pilot study, we investigated the presence of COL4A5 in the serum of patients with BOS.

**Methods:**

41 patients who had undergone LTX were enrolled. Of these, 27 developed BOS and 14 (control group) were considered stable at the time of serum sampling. Of BOS patients, serum samples were analysed at the time of BOS diagnosis and before the clinical diagnosis (pre-BOS). COL4A5 levels were detected through the ELISA kit.

**Results:**

Serum concentrations of COL4A5 were higher in pre-BOS than in stable patients (40.5 ± 13.9 and 24.8 ± 11.4, respectively, *p* = 0.048). This protein is not influenced by comorbidities, such as acute rejection or infections, or by therapies. Survival analysis also reveals that a higher level of COL4A5 was also associated with less probability of survival. Our data showed a correlation between concentrations of COL4A5 and FEV1 at the time of diagnosis of BOS.

**Conclusion:**

Serum concentrations of COL4A5 can be considered a good prognostic marker due to their association with survival and correlation with functional parameters.

**Supplementary Information:**

The online version contains supplementary material available at 10.1007/s00408-023-00632-8.

## Introduction

Lung transplant (LTX) is a life-saving treatment option for patients with severe chronic lung diseases [1]. Around 50% of transplanted patients develop chronic lung allograft dysfunction (CLAD) within 5 years [2]. Bronchiolitis obliterans syndrome (BOS) is the most common form of CLAD and is characterized by airflow limitation and an obstructive spirometric pattern without high-resolution computed tomography (HRCT) evidence of parenchymal opacities [3, 4]. In particular, computed tomography (CT) and microCT analysis show abundant small-airway obstruction, starting from the fifth generation of airway branching and affecting up to 40–70% of airways [5]. Histological analysis shows severe mononuclear infiltration of the airways, vessels, and septum, associated with fibrosis [6, 7].

The pathogenesis of BOS remains unclear. It is a multifactorial syndrome that leads to pathological tissue changes and is associated with the worst long-term survival in LTX patients [8, 9]. For all these reasons, there have been attempts to identify markers for early diagnosis of BOS [10, 11].

The main trigger of BOS is a fibro-proliferative process called the epithelial–mesenchymal transition, derived from self-perpetuating activation of fibroblasts and massive release of collagen leading to peribronchiolar fibrosis and a decline in lung function [12, 13].

The protein signature of BOS lesions concerns extracellular matrix organization, wound healing, and aberrant basement membrane composition. Basement membrane components, such as epitopes on collagens and laminins, are targeted by auto-antibodies and lymphocytes in bronchiolitis obliterans syndrome arising after lung transplant [13, 14].

The main protein involved in the alteration of basement membrane structure is collagen [15]. Thickening of the lamina reticularis impairs airway distensibility and patency [16]. Collagen types IV and VI are two proteins abundant in basement membrane; their loss of integrity causes abnormal lung architecture followed by fibrosis [13]. Collagen types I and V are two collagens of the lamina reticularis, a thin layer below the basement membrane [17, 18]. They are network-forming collagens that assemble into sheet-like networks and underlie the epithelial and endothelial cells [19, 20]. Mature collagen IV is a heterotrimer comprised of a combination of two or three of the six known collagen IV alpha chains which are increasingly recognized as being tissue- and organ-specific [16, 21]. Silencing of COL4 alpha 5 chain (COL4A5) is reported to impair endothelial cell barrier integrity and to alter tight junction formation [22]. In this pilot study, we investigated the presence of COL4A5 in serum of patients with BOS. The protein was detected and could be a useful predictive marker of BOS.

## Materials and Methods

### Study Design and Population

In this single-centre retrospective study, we enrolled 41 patients who had undergone LTX at the Lung Transplant Centre of Siena University Hospital between 2012 and 2022 and from whom serum samples were available. Of these, 27 developed BOS and 14 (control group) were considered stable at the time of serum sampling.

For all patients, demographic data, age of transplant, type of transplant, and the pathologies that made transplant necessary were recorded. For BOS patients, days of CLAD-free survival, BAL cell count, and lymphocyte subsets were recorded together with therapy at onset of BOS, previous episodes of acute rejection, and infections. BOS was graded following ISHLT guidelines [23].

Lung function tests (LFT), including FEV1, FVC, and DLCO percentages, as well as serum samples from the BOS group were obtained at and 3–6 months before diagnosis of BOS and also at the moment of BOS diagnosis[24]. At the time of serum sampling, patients were negative for infection or acute rejection. Patients with concurrent infections at the time of serum samples were excluded from the study.

### COL4A5 Detection

Serum concentrations of COL4A5 were measured by commercially available enzyme-linked-immuno-sorbent assay (ELISA kit, MyBioSource Inc) following the manufacturer’s instructions. Concentrations were read at 450 nm with a Victor X4 fluorimeter (Perkin Elmer Inc). Concentrations of COL4A5 were expressed in nanograms per millilitre (pg/ml).

### Statistical Analysis

Means and standard deviations (*M* ± SD) or medians and quartiles (25th and 75th percentiles) for continuous variables were used. A non-parametric one-way ANOVA test (Kruskal–Wallis test) and Dunn test were performed for comparisons of more than two groups. The Shapiro–Wilk test was used to test normal distribution of the variables. The Chi-squared test was used for categorical variables. Receiver-operating characteristic (ROC) curve analysis with areas under curves (AUC) was performed to detect the best cut-off values for sensitivity and specificity. The Youden index [*J* = max (sensitivity + specificity − 1)] was used to establish the best cut-offs for diagnosis. The Spearman test was used to look for correlations among variables. We also performed binomial and multinomial logistic regression to identify the variable that most influenced serum concentrations of COL4A5 and airway restriction severity. Kaplan-Meyer analysis was performed to associate serum concentrations of COL4A5 with overall survival of the entire cohort and CLAD-free days of BOS patients. Statistical analysis and graphic representation of data were performed by GraphPad Prism 9.0 software (GraphPad Holdings, LLC, San Diego, CA, USA) and Jamovi Free software. A *p* value less than 0.05 was considered statistically significant.

## Results

### Cohort Description

The main clinical characteristics are reported in Table 1. No differences in terms of age and type of transplant were found (*p* > 0.05). A predominance of males emerged in the BOS group. COPD/Emphysema and IPF were the two diseases most frequent in our cohort before transplant. Regarding outcome, 32.4% of BOS patients died, whereas members of the stable group were all alive on 31 December 2022. In the BOS group, the mean number of CLAD-free days was 1073 ± 1007.32, with a mean of survival after transplant of 2111.52 ± 1255.88 days. 22 (81.4%) patients showed almost one infection during follow-up and before the development of BOS. Impaired lung function was observed in BOS. At the time of diagnosis of BOS, 24 patients were on tacrolimus and 22 on azithromycin. Less than 50% of patients were on cyclosporin and/or mycophenolate therapy. Regarding BAL analysis performed at the time of serum sampling, an increase in neutrophil count (28.91 ± 32.13) with respect to normal reference values (< 1) was recorded. An elevated CD8 + T cell count (63.24 ± 12.8) and a lower than normal CD4/CD8 ratio (0.68 ± 1.2) were recorded.

**Table 1:** Demographic and clinical data of BOS and stable group

	BOS (*n* = 27)	Stable LTX (n = 14)	*p* value
SEX (M/F) (%m)	18/9 (66.5%)	5/9 (35.7%)	.01
Age (mean ± SD)	50.45 ± 13.48	52.42 ± 7.75	ns
Type of LTX (mono-lateral/bilateral)	10/17	2/12	.002
Diseases (n)			ns
COPD/Emphysema	7	4	
IPF	10	6	
CF	8	2	
HX	1	1	
FPD	1	0	
HP	1	1	
Survival (n.died/n.live)	12/15	0/14	< .0001
CRP (mg/dl)	0.22 ± 0.13	0.32 ± 0.19	ns
Previous acute rejection (yes/no)(%yes) If yes	12/15 (44%)	6/8 (42%)	ns
A1 (n)	4	4	
A2 (n)	4	1	
A3 (n)	4	1	
PFTs (%) before BOS diagnosis:			
FEV1	67.14 ± 14		
FVC	77.7 ± 16		
DLCO	59.3 ± 18		
PFTs (%) at BOS diagnosis:			
FEV1	54.4 ± 19		
FVC	70.2 ± 14		
DLCO	55.2 ± 14		

### Serum Concentrations of COL4A5

**Fig. 1:**
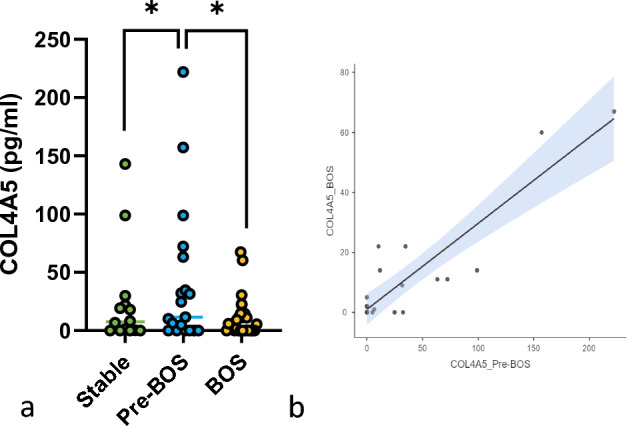
**a** COL4A5 pg/ml in the groups of the study b) Correlation of COL4A5 pre-BOS and at the moment of BOS diagnosis

Serum concentrations of COL4A5 were higher in pre-BOS than in stable patients (40.5 ± 13.9 and 24.8 ± 11.4, respectively, *p* = 0.048), and lower in pre-BOS than BOS patients (40.5 ± 13.9 and 10.6 ± 3.34, respectively, *p* = 0.039) (Fig. 1a). Moreover, a direct correlation was found between the serum concentrations of COL4A5 pre-BOS and at the time of BOS diagnosis (*r* = 0.89 *p* = 0.001) (Fig. 1b).

### Survival Analysis

ROC curve analysis of BOS and stable patients did not discriminate the two groups (*p* > 0.05); a cut-off value of 5.64 pg/ml was the best concentration in terms of specificity and sensitivity (57.14 and 55.56%, respectively). Comparing pre-BOS concentrations of COL4A5 and stable patients, the best cut-off point was 10.82 pg/ml with a specificity and sensitivity of 57.14 and 52.6%, respectively.

**Fig. 2:**
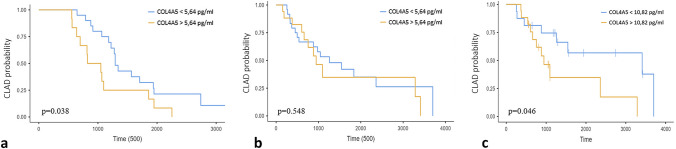
**a** Kaplan–Meier survival probabilities curves in LTX patients (41 patients) stratify based on COL4A5 concentrations (< 5.64 pg/ml). **b** Kaplan–Meier CLAD-free survival curves of patients with BOS stratify based on COL4A5 concentrations (< 5.64 pg/ml). **c** Kaplan–Meier CLAD-free survival curves of patients with BOS stratify based on COL4A5 concentrations before BOS concentrations (10.82 pg/ml)

When our entire cohort was stratified on the basis of serum concentrations of COL4A5 (< 5.64 pg/ml and > 5.64 pg/ml), the former showed a higher probability of survival (*p* = 0.038) (Fig. 2a).

No statistically significant difference in number of CLAD-free days emerged between the two groups (*p* = 0.548) (Fig. 2b).

When we stratified our BOS cohort on the basis of a pre-BOS COL4A5 cut-off value of 10.82 pg/ml (< 10.64 pg/ml and > 10.82 pg/ml), we observed that the former group had longer CLAD-free survival than the latter group (*p* = 0.046) (Fig. 2c).

### Multinomial Logistic Regression

The logistic regression models were designed to explore the role of predictors that may affect serum concentrations of COL4A5. In the first two models, we performed binomial logistic regression and assigned our cohort scores of 0 and 1 on the basis of the two cut-offs (5.64 pg/ml and 10.82 pg/ml), using survival, type of transplant, acute rejection, infections, and therapies as intercepts. None of these variables influenced stratification of the groups (Suppl. Tables 1–2).

A third model was built using the two groups (Stable and BOS) as coefficients. In this case, serum COL4A5 (*z* score: 2.27, *p* = 0.02), COL4A5 pre-BOS (*z* score: − 2.17, *p* = 0.03), and overall survival (*z* score: 1.85, *p* = 0.06) influenced the development of BOS (Table 2). This means that BOS development impacts on COL4A5 levels both on COL4A5 at the moments of diagnosis and 3–6 months before clinical diagnosis.

**Table 2:** Logistic regression model using as dependent variable, the two groups of analysis: Stable and BOS

Model coeffecients group				
Predictor	Estimate	SE	*z*	*p*
Intercept	– 3.42437	1.73699	– 1.97	0.049
Col4a5preBOS	– 0.10389	0.04786	– 2.17	0.030
COL4AS	0.19064	0.08398	2.27	0.023
Overall survival days	0.00238	0.00129	1.85	0.065

Estimate represents the log odds of “groups = stable” vs. “groups = BOS”

### Correlation Analysis

**Fig. 3:**
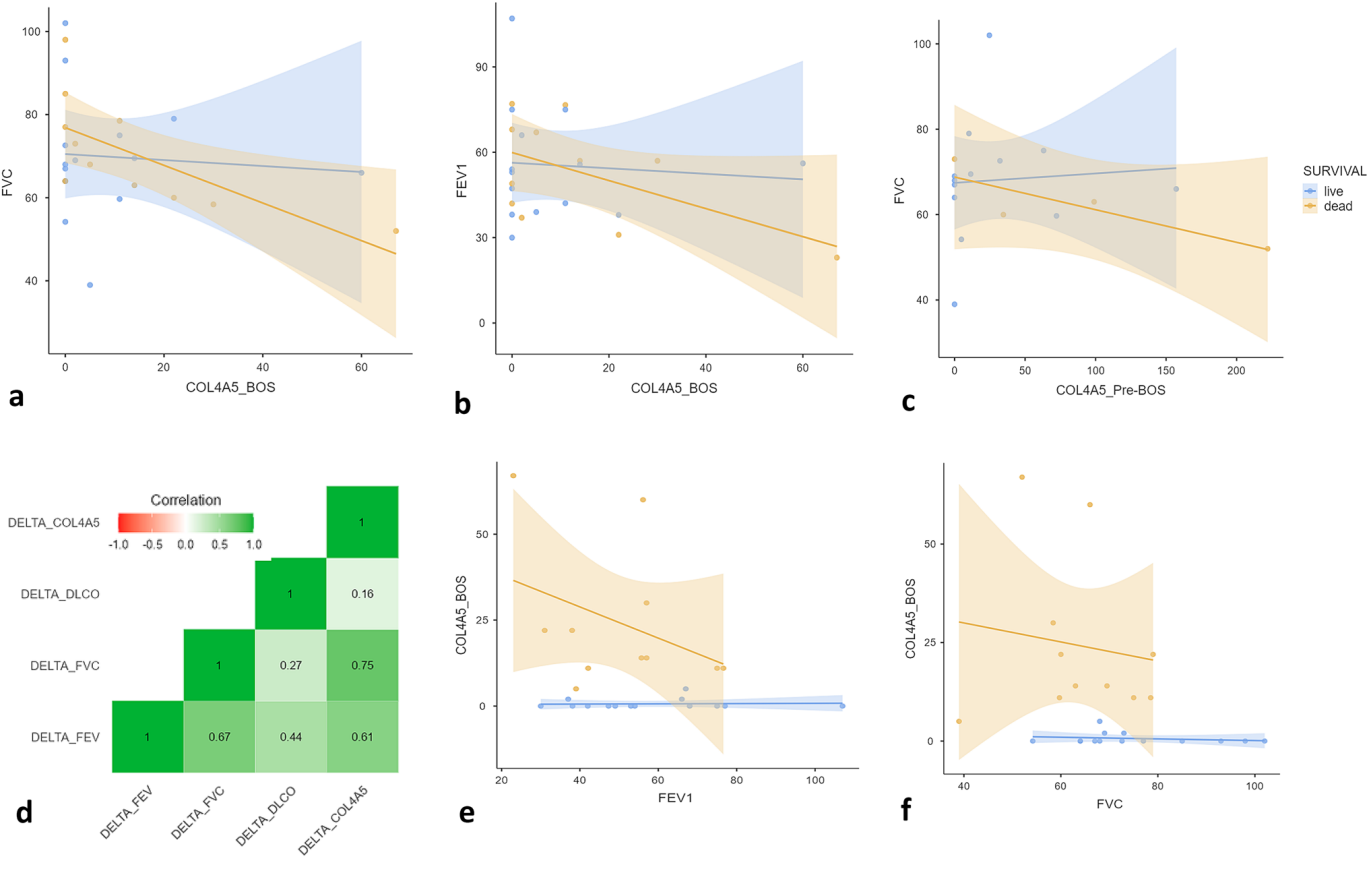
**a** Spearman correlation analysis of FVC and COL4A5 concentrations at BOS diagnosis in patients stratifies as live and dead. **b** Spearman correlation analysis of Fev1 and COL4A5 concentrations at BOS diagnosis in patients stratifies as live and dead. **c** Spearman correlation analysis of FVC and COL4A5 concentrations pre-BOS diagnosis in patients stratifies as live and dead. **d** Δ correlation analysis between pulmonary functions and COL4A5 levels. **e**, **f** Spearman correlation analysis of FEV1 (**e**) and FVC (**f**) and COL4A5 concentrations at BOS diagnosis in patients stratifies as COL4A5 < 5.64 and COL4A5 > 5.64 pg/ml

Figure 3a–c reports the correlation between functional parameters (FEV1, FVC, and DLCO expressed as percentages) and COL4a5 concentration at the moment of BOS diagnosis. Moreover patients were stratified for survival data (as live and dead).

This stratification showed an inverse correlation between serum concentration of COL4A5, predicted FVC (Fig. 3a) (*r* = − 0.56 *p* = 0.03), and FEV1 (Fig. 3b) (*r* = − 0.42 *p* = 0.04) at the time of diagnosis of BOS and reached particular statistical significance for patients who died.

When serum concentrations of COA4A5 pre-BOS were analysed, only FVC proved to be statistically significant for patients of the BOS group who died (*r* = − 0.52; *p* = 0.04) (Fig. 3c).

Differences (Δ) between serum concentrations of COL4A5 before and at diagnosis of BOS were calculated. ΔCOL4A5 showed a significant correlation with ΔFEV1 (*r* = 0.61 *p* = 0.005) and ΔFVC (*r* = 0.75 *p* = 0.003) but not with DLCO (Fig. 3d).

After the stratification of patients into two groups (group 0 with COL4A5BOS < 5.64 pg/ml and group 1 with [COL4A5]BOS > 5.64 pg/ml), an inverse correlation with FEV1 (*r* = − 0.65 *p* = 0.001) (Fig. 3e) and FVC (*r* = − 0.31 *p* = 0.048) (Fig. 3f) only emerged in the case of group 1.

## Discussion

Bronchiolitis obliterans syndrome is a common complication of lung transplant and is a negative event for survival. Criteria for the diagnosis of BOS are defined by ISHLT guidelines [23] and include clinical and functional parameters such as FEV1, FVC, and DLco. Although the histological features of BOS, especially in the later stages of the disease, have been abundantly described, early diagnosis of BOS is still a challenge. For this reason, many studies have focused on biomarkers that could help clinicians predict patients at higher risk of developing BOS. Surveillance bronchoscopy, including bronchoalveolar lavage and transbronchial biopsy, is part of routine follow-up and involves invasive procedures often inadvisable in patients in poor condition. This is why we chose serum as a safe, readily obtained, and cost-effective biological matrix.

We investigated the role of serum concentrations of COL4A5 as a potential marker of BOS. Our results showed that already 3–6 months before diagnosis of BOS, peripheral concentrations of COL4A5 were higher in patients who developed BOS than in stable patients.

Serum concentrations of COL4A5 can also be considered a good prognostic marker due to their association with survival, being lower in survivors than in patients who died. In the deceased group, a correlation was demonstrated between the marker and impaired lung function. Interestingly, this protein is not influenced by comorbidities, such as acute rejection or infections, or by therapies for BOS.

From a biological point of view, the main biological triggers of BOS remain unclear. Self-perpetuating activation of fibroblasts and excessive release of collagen leading to peribronchiolar fibrosis after a decline in lung function and airway obstruction are reported to be associated with onset of BOS [25]. This led to research into several markers related to the extracellular matrix (ECM), such as matrix metalloproteinase-9 (MMP-9), and to tissue remodelling, such as VEGF and VEGF receptor 2 (VEGFR2) [26–28].

In particular, in COPD patients, ECM turnover may damage lung architecture, impairing lung function [16]. Matrix metalloproteinase breaks down different fragments of collagen. While patients chronic rejection showed small-airway inflammation known as “obliterative bronchiolitis” that leads to epithelial damage, airway fibrosis and remodelling of the ECM affect the lung interstitial matrix and basement membrane [29]. Some studies showed that besides their role in tissue remodelling, serum concentrations of different MMPs (especially MMP-9) were correlated with BAL neutrophilia in patients with BOS [30, 31]. Interestingly, our cohort of patients also showed BAL neutrophilia.

It has also been demonstrated that FEV1 percentages are inversely correlated with collagen concentration on the surface of the epithelial basement membrane, suggesting that increased bronchial deposition of collagen contributes to deteriorating lung function and airway remodelling [16]. In line with this finding, our data showed a correlation between serum concentrations of COL4A5 and FEV1 at the time of diagnosis of BOS.

The limits of our study include its monocentric and retrospective nature. However, it is the first study to investigate serum concentrations of COL4A5 in BOS patients at and before clinical diagnosis of BOS, and to propose COL4A5 as a potential predictive biomarker of BOS development and as a marker of negative prognosis in lung transplant patients.

## Supplementary Information

Below is the link to the electronic supplementary material.

Supplementary file1 (DOCX 113 kb)
