# Supplementary material for: Collagen Type IV Alpha 5 Chain in Bronchiolitis Obliterans Syndrome After Lung Transplant: The First Evidence
Source: Lung. 2023 Jul 4;201(4):363–9. doi: 10.1007/s00408-023-00632-8 (PMC10444639; doi:10.1007/s00408-023-00632-8)
Supplement: Supplementary file 1 — Supplementary file1 (DOCX 113 kb) [file 408_2023_632_MOESM1_ESM.docx]

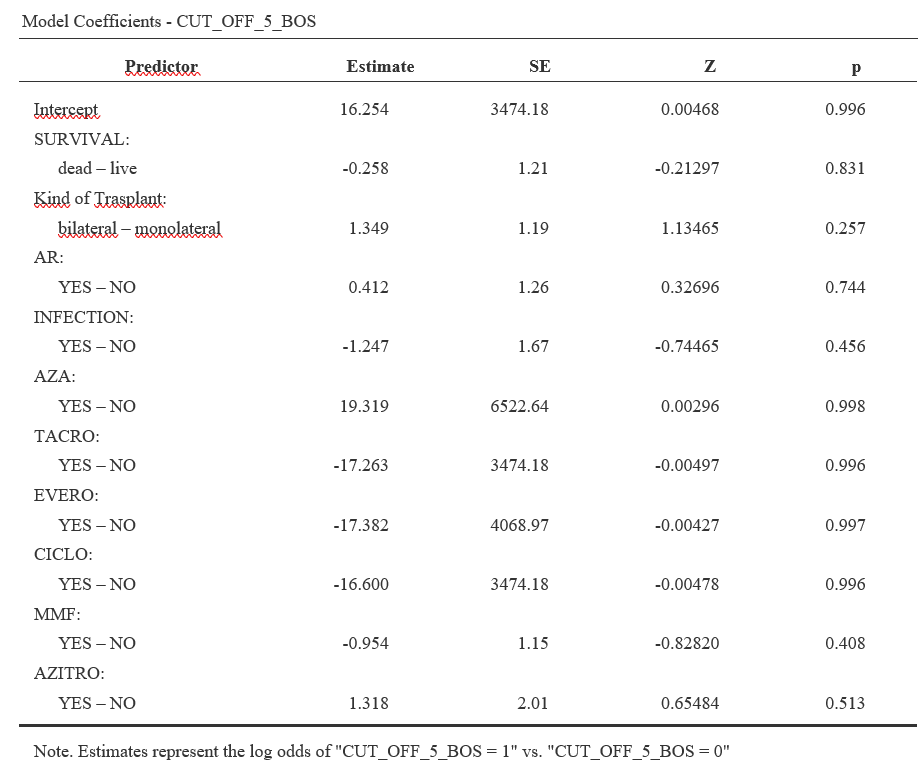


**Suppl.Tab 1** Binomial logistic regression with 5.64 pg/ml as model coefficients.


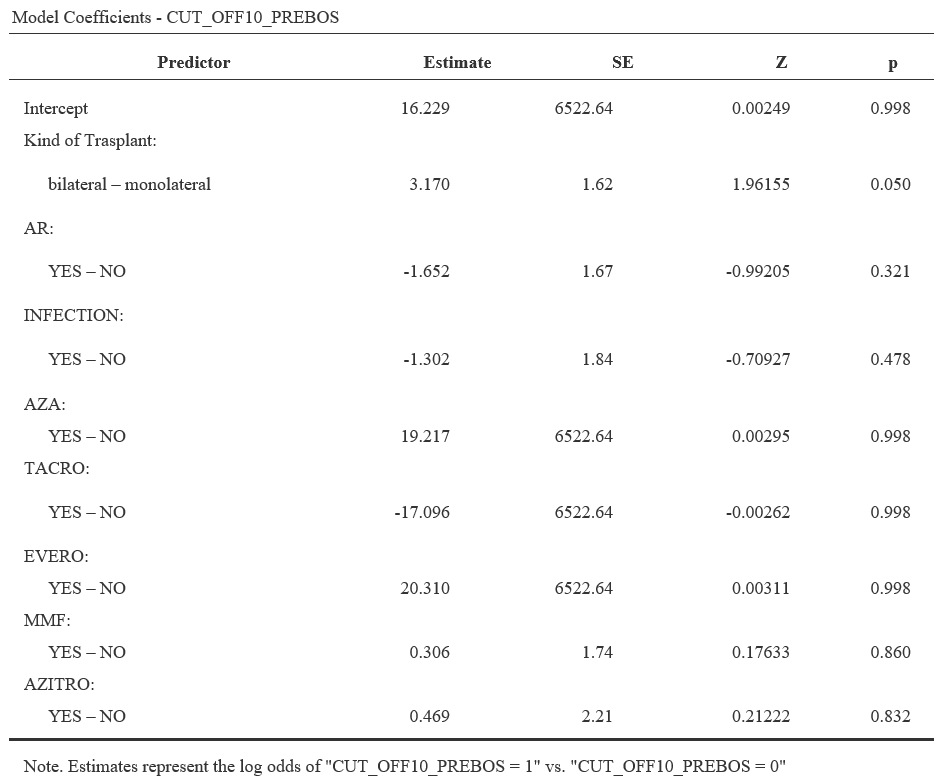


**Suppl.Tab 2** Binomial logistic regression with 10.82 pg/ml as model coefficients
